# The Impact of Palivizumab for Respiratory Syncytial Virus Prophylaxis on Preschool Childhood Asthma

**DOI:** 10.3390/vaccines12111269

**Published:** 2024-11-10

**Authors:** Hannah Ora Hasson, Yoav Bachar, Itai Hazan, Inbal Golan-Tripto, Aviv Goldbart, David Greenberg, Guy Hazan

**Affiliations:** 1 The Faculty of Health Sciences, Ben-Gurion University of the Negev, Beer Sheva 8410501, Israelinbal_gt@yahoo.com (I.G.-T.);; 2 Pediatric Pulmonary Unit, Saban Children’s Hospital, Soroka University Medical Center, Beer Sheva 8400101, Israel; 3 Pediatric Infectious Diseases Unit, Saban Children’s Hospital, Soroka University Medical Center, Beer Sheva 8400101, Israel

**Keywords:** asthma, respiratory syncytial virus

## Abstract

Background: The respiratory syncytial virus (RSV) is a leading cause of lower respiratory tract infections in infants and is associated with an increased risk of asthma development. Palivizumab, an RSV prophylactic, reduces RSV-related hospitalizations in high-risk infants, but its impact on long-term asthma outcomes remains unclear. This study compares asthma-related healthcare utilization in preschool children born prematurely between those who received Palivizumab (the Prophylaxis (+) group) and those who did not (the Prophylaxis (–) group). Methods: This nationwide, population-based retrospective cohort study utilized data from Clalit Healthcare Services in Israel. The study included children born between 32 + 6 and 34 + 6 weeks of gestational age from 2011 to 2018. Descriptive analysis, univariate analysis, and multivariate logistic regression were performed to compare the Prophylaxis (+) and the Prophylaxis (–) groups. Results: In total, 4503 children were included, with 3287 in the Prophylaxis (+) group and 1216 in the Prophylaxis (–) group. Palivizumab administration was associated with reduced hospitalizations for RSV bronchiolitis (1.8% vs. 3.3%, *p* = 0.003). However, no significant differences were observed in multivariate analysis for long-term asthma outcomes, including asthma diagnosis (OR = 1.04, CI = 0.84–1.30, *p* = 0.7) or emergency department visits for asthma (OR = 0.79, CI = 0.54–1.17, *p* = 0.2). Similarly, Palivizumab administration was not associated with the purchase of short-acting beta-agonists (OR = 1.14, 95% CI 0.98–1.32, *p* = 0.084), inhaled corticosteroids (OR = 1.1, CI = 0.93–1.32, *p* = 0.3), or oral corticosteroids (OR = 1.09, CI = 0.94–1.26, *p* = 0.3). Conclusions: While Palivizumab effectively reduces RSV acute bronchiolitis in preterm infants, it does not significantly impact long-term preschool asthma-related healthcare utilization.

## 1. Introduction

The respiratory syncytial virus (RSV) is the primary cause of lower respiratory tract infections in children under two years of age [1,2]. Numerous studies have consistently found a link between RSV infection during infancy and the later development of childhood asthma [3,4,5,6]. Acute RSV infection can result in the immune cell infiltration of the airways, triggering a heightened proinflammatory response and causing significant lung damage [1,7]. This inflammation may lead to chronic respiratory morbidities, especially if it occurs in premature infants [8]. 

The interplay between RSV infections in early life and asthma development is not yet fully understood. One theory suggests that lung damage or modifications in epithelial and airway reactivity caused by RSV-induced bronchiolitis predispose individuals to asthma development [9]. The second theory argues that RSV infection is a marker for asthma predispositions rather than a causative agent [10,11]. Factors such as RSV type, genetics, environment, and the immune system play complex roles in RSV and asthma development. Therefore, to determine possible causality, it is necessary to investigate the impact of RSV prevention on the development of childhood asthma [12].

Palivizumab is a humanized immunoglobulin-1 monoclonal antibody designed to target a conserved epitope of the fusion (F) protein in all RSV serotypes, preventing RSV from entering airway epithelial cells and causing injury. In many high-income countries, this therapeutic agent is administered to high-risk infants to prevent RSV acute bronchiolitis [2,13]. Since 2014, this intervention has been licensed in Israel for use in late premature infants (gestational age of 32 + 6 to 34 + 6 weeks) [14]. Previous studies have shown that Palivizumab administration reduces wheezing in the first years of life and reduces hospitalization from RSV infection in high-risk infants [5,13,14,15,16]. However, data on its long-term effects on asthma development are limited. With the emergence of new RSV vaccines, understanding their long-term impact on healthcare utilization for asthma is particularly important. 

This study aims to utilize a nationwide cohort to compare preschool asthma-related healthcare utilization in children with a history of prematurity who received the monoclonal antibody Palivizumab (Prophylaxis (+) group) with those who did not (Prophylaxis (–) group). By exploring the interplay between RSV prophylaxis, RSV infection, and the development of childhood asthma, this research offers valuable insights into the pathogenesis of asthma inception and provides essential perspectives on the expected long-term impact of upcoming RSV vaccines. 

## 2. Methods 

### 2.1. Study Design and Setting

This population-based retrospective study utilizes a nationwide computerized Clalit Healthcare Services (CHS) database to compare asthma-related healthcare utilization among children born between 1 January 2011 and 31 December 2018 at a gestational age of 32 + 6 to 34 + 6 weeks. CHS, Israel’s largest state-mandated not-for-profit healthcare provider, serves over 5 million members, representing 52% of the population. The CHS database includes comprehensive demographic data, anthropometric measurements, community clinic and hospital diagnoses, medication dispensing information, and laboratory results. All data were de-identified before analysis, and the study involved the secondary use of existing clinical information. 

The study was approved by the CHS ethical committee (Helsinki number 0193-23-SOR). In Israel, the eligibility criteria for Palivizumab were expanded in 2014 to include infants born at less than 34 + 6 weeks of gestational age, in addition to those born before 32 + 6 weeks. This expansion enabled a comparison between premature infants who received the Palivizumab vaccination and those who did not. The case group (Prophylaxis (+) group) included children born after 2014 with documented monoclonal antibody (Palivizumab) administration in their medical records, while the control group (Prophylaxis (–) group) comprised children born before 2014 without documentation of Palivizumab administration. Notably, only infants born between July and December each year since 2014 who would be under six months old during the RSV season were eligible for Palivizumab administration in this gestational age group [17]. 

### 2.2. Study Population

The analysis included all CHS members born between 32 + 6 and 34 + 6 weeks of gestational age from 1 January 2011 to 31 December 2018. The asthma-related outcomes measured were analyzed between 1 and 5 years of age. Children with chromosomal abnormalities, congenital lung disease unrelated to prematurity, congenital heart disease, or primary immunodeficiency were excluded from the analysis (see Table S1). 

### 2.3. Data Sources and Organization

We analyzed de-identified patient-level data extracted from CHS electronic medical records (MDCLONE system). This dataset comprised information such as date of birth, sex, the diagnosis of allergic rhinitis, atopic dermatitis, food allergies, mode of delivery, gestational age, birth weight, the presence of multiple births, and eosinophil counts collected between the ages of 6 and 18 months. Additionally, we obtained data on maternal asthma history by linking the child’s file to the mother’s chart. Socioeconomic status (SES) was also included, utilizing each member’s enumeration area of residence as reported by the Israeli Central Bureau of Statistics and Points Business Mapping Ltd.© 32 (Population Data by City, Israel). 

### 2.4. Study Outcomes

ICD-9 codes (see Table S1) were used to identify asthma or wheezing diagnoses as primary outcome measures. RT-qPCR results for RSV and ICD codes for RSV acute bronchiolitis were extracted, as RT-qPCR testing is only available in hospital settings. The results were obtained from routine respiratory panels conducted as part of clinical services. Regarding laboratory tests for RSV detection as part of the routine protocol of the pediatric division at the SUMC, all patients with suspected respiratory infection symptoms were evaluated for respiratory viruses. Nasopharyngeal wash specimens were obtained within 48 h of admission from hospitalized children during working hours (excluding weekends and holidays as described elsewhere) [18]. Only one specimen from each patient was included. Nasopharyngeal wash specimens, obtained using a 2 × 10^5^ mL of 0.9% saline solution with a mucus extractor kit (Maersk A/S, Lynge, Denmark), were sent to the Clinical Virology Laboratory within 6 h [19]. Respiratory viruses were detected by nucleic acid extraction using a NucliSens EasyMag apparatus (BioMérieux, Marcy l’Etoile, France), following the manufacturer’s instructions. The sets of primers and probes for detecting 12 viruses via multiplex hydrolysis probe-based quantitative real-time reverse transcription PCR (RT-PCR) have been described previously [20]. Each sample was tested in parallel, using three tubes, for the following viruses: influenza A and B; parainfluenza virus types 2 and 3; human respiratory syncytial virus (RSV); human metapneumovirus (hMPV); rhinovirus; adenovirus; and coronaviruses 229E, HKU1, OC43, and NL63. Amplification was performed in a final volume of 10 μL using an RNA Ultrasense one-step quantitative real-time RT-PCR system (Invitrogen, Carlsbad, CA, USA) with 4 μL of nucleic acid, four sets of primers and probes to detect four viruses, and an internal control set. Amplification was conducted on a 7500 Real-Time PCR System thermocycler (Applied Biosystems, Foster City, CA, USA).

Given the complexity of diagnosing asthma in preschool-aged children, several proxies for asthma were analyzed, including emergency department (ED) visits or hospitalizations for asthma or wheezing (identified via ICD-9 codes), as well as purchases for short-acting beta-agonist (SABA) inhalers, inhaled corticosteroids (ICSs), and oral corticosteroids (OCSs). Medications prescribed to CHS members were purchased at pharmacies connected to a centralized database, allowing for monitoring through the CHS system. To enhance the specificity of the asthma diagnosis, a new variable, the “Asthma Integrated Diagnosis Index”, was defined. This index includes the following criteria: the use of SABAs over two different years within the first five years of life, at least one instance of ICS usage, and at least one ICD-9 code for asthma or wheezing from either community or hospital admissions. 

### 2.5. Statistical Analysis 

The initial descriptive analysis involved calculating the distribution, central tendency, and dispersion of single variables. Univariate analysis was conducted using the chi-square test for dichotomous variables, Student’s t-test for normally distributed continuous variables, and the Mann–Whitney U-test for categorical or non-normally distributed continuous variables. Following this, multivariate logistics and Poisson regressions were performed using the backward elimination method. In Israel, the RSV season is from autumn to spring. Premature babies born up to weeks 34 + 6 are administered RSV prophylaxis if they are not yet six months old when November begins in that same season. All children entitled to RSV prophylaxis continue to receive it until the end of the RSV season [17]. Owing to the impact of birth season on RSV infection risk and eligibility for RSV prophylaxis, a similar analysis was conducted exclusively on patients born between July and December each year [21,22]. 

## 3. Results 

**Figure 1:**
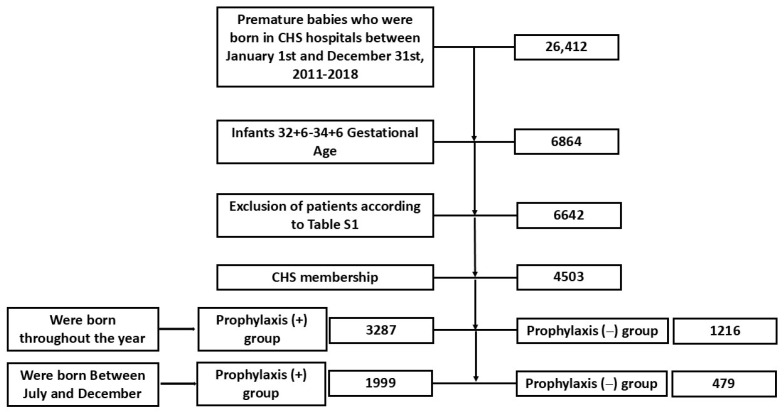
Flowchart depicting the selection process for premature infants included in this study. It outlines the number of premature babies born in CHS hospitals between January 1st and December 31st, 2011–2018, and incorporates the exclusion criteria noted in the Section 2. Demographic and clinical characteristics are detailed in Table 1. The distribution of male sex and Jewish ethnicity was similar between the Prophylaxis (–) and Prophylaxis (+) groups (56% vs. 54%, *p =* 0.3, and 93% vs. 93%, *p* = 0.7, respectively). The median gestational age was slightly earlier in the Prophylaxis (+) group than in the Prophylaxis (–) group (median = 33 weeks, IQR = 33–34 vs. median = 34 weeks, IQR = 33–34, *p* < 0.001). Birth weight was also significantly lower in the Prophylaxis (+) group than in the Prophylaxis (–) group (1979 ± 385 g vs. 2165 ± 470 g, *p* < 0.001). Cesarean deliveries were more common in the Prophylaxis (+) group than in the Prophylaxis (–) group (57% vs. 52%, *p* = 0.003).

Between 2011 and 2018, 26,412 premature babies were born in CHS hospitals (Figure 1). Of these, 6864 were born within the relevant gestational age range of 32 + 6 to 34 + 6 weeks. Among them, 222 infants were excluded because of chromosomal abnormalities or chronic lung diseases (see Table S1). Additionally, patients with incomplete medical chart follow-ups—resulting from having health insurance with a provider other than CHS—were also excluded, leaving 4503 children enrolled in this study. Of these, 3287 were in the Prophylaxis (+) group, and 1216 were included in the Prophylaxis (–) group.

Regarding the complications associated with premature birth, the Prophylaxis (+) group experienced more complications overall. Respiratory distress syndrome was significantly more frequent in the Prophylaxis (+) group (8.8% vs. 5.2%, *p* < 0.001). Similarly, intraventricular hemorrhage (IVH) and patent ductus arteriosus (PDA) were more common in the Prophylaxis (+) group (IVH: 2.3% vs. 1.1%, *p* = 0.009; PDA: 2.9% vs. 1.6%, *p* = 0.01). However, there were no statistically significant differences between the groups in the proportions of bronchopulmonary dysplasia (BPD) and necrotizing enterocolitis (NEC) diagnoses (0% vs. 0.3%, *p* = 0.12, and 0.7% vs. 0.9%, *p* = 0.4, respectively). 

Next, a univariate analysis was conducted to evaluate the clinical outcomes that may impact the development of asthma or serve as surrogates for asthma diagnosis in children aged 1–5 years (Table 2 and Table S2). The proportion of admissions for RSV bronchiolitis in the first two years of life was higher in the Prophylaxis (–) group than in the Prophylaxis (+) group (3.3% vs. 1.8%, *p* = 0.003). Outcomes indicative of atopy, including allergic rhinitis (OR = 0.99, CI = 0.69–1.43, *p* = 0.9), atopic dermatitis (OR = 1.11, CI = 0.95–1.29, *p* = 0.2), and mean absolute eosinophilic blood counts (IQR = 0.3–0.2, *p* = 0.1213) showed no significant differences between the Prophylaxis (+) and Prophylaxis (–) groups. Similarly, the proportions of ICD-9 codes for asthma, as well as emergency department visits for asthma or wheezing, were not significantly different between the groups (OR = 1.16, CI = 0.95–1.43, *p* = 0.14; OR = 0.81, CI = 0.58–1.16, *p* = 0.3, respectively). However, the proportion of SABA purchases was 19% higher in the Prophylaxis (+) group than in the Prophylaxis (–) group (CI = 1.04–1.36, *p =* 0.01). Additionally, the proportion of patients who met the criteria for the “Asthma Integrated Diagnosis Index” was 34% higher in the Prophylaxis (+) group than in the Prophylaxis (–) group (CI = 1.05–1.74, *p* = 0.04). 

**Figure 2:**
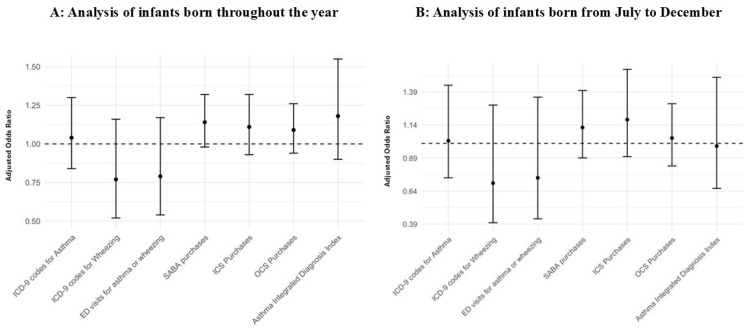
(**A**,**B**) Adjusted odds ratios from multivariate logistic regression analysis comparing healthcare utilization for asthma-related outcomes between Prophylaxis (+) and Prophylaxis (–) groups. The figure presents odds ratios (ORs) with 95% confidence intervals for various asthma-related outcomes. The dashed line represents an odds ratio of 1.0, indicating no difference between the groups. All ORs are adjusted for potential confounders such as birth weight, atopic dermatitis, mode of delivery, and absolute eosinophil count.

A multivariate analysis was subsequently conducted to assess the outcomes that serve as asthma surrogates, with adjustments made for atopic dermatitis, birth weight, mode of delivery, and absolute eosinophil counts (Figure 2A and Table S3). The analysis revealed no significant differences between the Prophylaxis (+) and Prophylaxis (–) groups in any of the asthma surrogates, including ICD-9 codes for asthma (OR = 1.04, 95% CI 0.84–1.30, *p* = 0.7) or wheezing diagnoses (OR = 0.77, 95% CI 0.52–1.16, *p* = 0.2) and ED visits for asthma or wheezing (OR = 0.79, 95% CI 0.54–1.17, *p* = 0.2). Similarly, vaccination showed no association with SABA purchases (OR = 1.14, 95% CI 0.98–1.32, *p* = 0.084), ICS purchases (OR = 1.1, CI = 0.93–1.32, *p* = 0.3), or OCS purchases (OR = 1.09, CI = 0.94–1.26, *p =* 0.3). Additionally, the Asthma-Integrated Diagnosis Index did not differ significantly between the two groups (adjusted OR = 1.16, CI = 0.9–1.51, *p* = 0.3).

Palivizumab was administered monthly throughout the RSV season during the cold months between September and January [7]. To account for Palivizumab’s seasonal timing and to minimize confounders, we conducted a sub-analysis excluding children born between January and June, as they were not eligible for the Palivizumab administration, being older than six months during the RSV season. The findings for this sub-cohort were similar to those for our primary cohort (Figure 2B and Table S4). Prophylaxis administration was not significantly associated with asthma diagnosis (OR = 1.02, 95% CI, 0.74–1.44, *p* = 0.9), ED visits for asthma or wheezing (OR = 0.70, 95% CI, 0.40–1.29, *p* = 0.3), wheezing diagnosis (OR = 0.71, 95% CI, 0.41–1.29, *p* = 0.2), SABA purchase (OR = 1.12, 95% CI, 0.89–1.40, *p* = 0.4), ICS purchase (OR = 1.18, 95% CI, 0.90–1.56, *p* = 0.2), or the Asthma-Integrated Diagnostic Index (OR = 0.98, 95% CI, 0.66–1.50, *p* > 0.9). 

## 4. Discussion

This study aimed to investigate the impact of Palivizumab, an RSV monoclonal antibody prophylactic, on the incidence of preschool asthma-related healthcare utilization in children born prematurely at less than 35 weeks of gestation. While Palivizumab significantly reduced the incidence of RSV acute bronchiolitis, there was no significant difference in long-term preschool asthma-related outcomes. Specifically, there was no significant difference in the incidence of asthma diagnosis, ED visits for asthma or wheezing, or the purchase of asthma medications such as ICS or OCS. Notably, while the proportion of SABA purchases was higher in the Prophylaxis (+) group in the univariate analysis, after adjusting for potential confounders, the trend of no significant differences between the groups remained consistent. This was evident in the multivariate logistic regression and sub-cohort analyses by birth month. The Prophylaxis (+) group had significantly earlier gestational ages at birth; lower birth weights; and a higher incidence of respiratory distress syndrome, cesarean delivery, and certain congenital heart defects. This may explain the higher SABA usage observed in this group in the univariate analysis.

Palivizumab significantly reduced the incidence of hospitalizations for RSV acute bronchiolitis in the Prophylaxis (+) group compared with the Prophylaxis (−) group in the first two years of life (1.8% vs. 3.3%, *p* = 0.003). This aligns with previous studies showing reduced RSV-related hospitalizations and wheezing episodes during the first year of life following Palivizumab administration [5,13]. A randomized controlled trial investigating the association between RSV prophylaxis and asthma found a lower rate of wheezing at age 1 in the vaccinated group [15]. However, a follow-up study at age 6 found no significant differences between vaccinated and non-vaccinated groups regarding physician-diagnosed asthma or FEV0.5 [23]. These findings are consistent with the current study, which demonstrated the acute beneficial effect of vaccination in preventing wheezing episodes but no long-term effect. A prospective case–control study conducted in Japan highlighted the significant impact of RSV prevention on reducing recurrent wheezing episodes at age six. However, no noticeable effect was observed on the development of atopic asthma [15]. Subsequent research has been limited by small sample sizes and reliance on subjective reports of wheezing as an indicator of asthma [10,12,13,24,25,26]. The present study aimed to overcome these weaknesses by incorporating more objective measures of healthcare utilization, using a nationwide large cohort, and adjusting for atopy and a family history of asthma.

Several limitations should be considered. First, the retrospective design of this study presents inherent constraints. Relying on healthcare utilization records to identify asthma and wheezing may miss some cases, particularly milder forms managed without formal medical intervention. Additionally, the burden of RSV in community settings could not be assessed, as RSV RT-qPCR data were only available for hospitalized patients, limiting insight into the broader impact of RSV outside of hospital admissions. In studies on early childhood asthma, defining the condition is often unclear and debated. To address this, several asthma-related outcomes were used, and an “Integrated Asthma Diagnosis Index” was created to standardize the asthma cases. 

In summary, this retrospective study examined the impact of Palivizumab on preschool asthma development in preterm infants. While Palivizumab effectively reduces the incidence of RSV-related acute bronchiolitis, it does not appear to influence the long-term risk of preschool asthma or wheezing. In this context, it is important to note that Palivizumab is a passive immunization delivered as a monoclonal antibody, and its effectiveness may diminish over time as antibody levels wane. This may partly explain the limited long-term impact observed. Understanding the strengths and limitations of the RSV vaccine is crucial, particularly as new RSV vaccines are developed and evaluated [27,28]. While the short-term benefits of Palivizumab are evident in reducing the incidence of acute RSV bronchiolitis in premature infants, this study’s findings suggest that its long-term benefits may be limited. Instead, we suggest that early-life RSV infection may reflect an underlying predisposition to asthma rather than serving as a direct cause, as preventive interventions have not reduced asthma incidence. This creates a “natural experiment” scenario, underscoring the need for further research into the relationship between early-life RSV infections, preventive strategies, and long-term childhood preschool asthma.

## 5. Conclusions

This study demonstrates that while the monoclonal antibody Palivizumab effectively reduces RSV-related acute bronchiolitis in preterm infants, it does not significantly influence long-term preschool asthma-related outcomes, such as asthma diagnosis, emergency department visits for asthma or wheezing, or the use of asthma medications. These findings confirm Palivizumab’s short-term benefits in decreasing RSV hospitalizations but suggest its limited impact on long-term asthma development. This underscores the necessity for further research on RSV infections and preventive strategies, particularly with forthcoming new vaccine modalities, to better understand their connection to childhood asthma.

## Figures and Tables

**Table 1:** Demographic and clinical variables—descriptive analysis comparing Prophylaxis (+) and Prophylaxis (–) groups.

Characteristic	Prophylaxis (–) N = 1216	Prophylaxis (+) N = 3287	*p*-Value
Male sex, N (%)	682 (56%)	1790 (54%)	0.3
Jewish ethnicity, N (%)	1130 (93%)	3042 (93%)	0.7
Median birth week (IQR) *	34.00 (33.00, 34.00)	33.00 (33.00, 34.00)	<0.001
Mean birth weight (±SD) **	2165 ± 470 (1216)	1979 ± 385 (3287)	<0.001
Mean maternal age (±SD) **	30.7 ± 6.0 (1216)	31.0 ± 6.0 (3287)	0.12
Cesarean delivery, N (%)	637 (52%)	1885 (57%)	0.06
Low socioeconomic score, N (%)	305 (27%)	825 (28%)	0.6
Diagnosis of bronchopulmonary dysplasia, N (%)	0 (0%)	9 (0.3%)	0.12
Diagnosis of respiratory distress syndrome, N (%)	63 (5.2%)	290 (8.8%	<0.001
Diagnosis of necrotizing enterocolitis, N (%)	8 (0.7%)	31 (0.9%)	0.4
Diagnosis of intraventricular hemorrhage of the fetus, N (%)	13 (1.1%)	75 (2.3%)	0.009
Patent ductus arteriosus, N (%)	19 (1.6%)	96 (2.9%)	0.01

Unless otherwise stated, the statistical test used for comparisons between the two groups was the chi-square test. * Comparisons were performed using the Mann–Whitney test. ** Comparisons were conducted using Student’s *t*-test.

**Table 2:** Univariate analysis of clinical outcomes between the Prophylaxis (+) and Prophylaxis (–) groups at 1–5 years of age.

Clinical Outcome	Prophylaxis (–) N = 1216	Prophylaxis (+) N = 3287	*p*-Value	Odds Ratio (95% Confidence Intervals)
Admission for RSV bronchiolitis *, N (%)	40 (3.3%)	60 (1.8%)	0.004	0.55
				(0.37–0.83)
Allergic rhinitis, N (%)	42 (3.5%)	112 (3.4%)	0.9	0.99
				(0.69–1.43)
Atopic dermatitis, N (%)	289 (24%)	843 (26%)	0.2	1.11
				(0.95–1.29)
Mean absolute eosinophilic blood ** (±SD)	0.28 ± 0.22	0.3 ± 0.28	0.12	-
ICD-9 codes for asthma diagnosis, N (%)	141 (12%)	435 (13%)	0.14	1.16
				(0.95–1.43)
ICD-9 codes for wheezing diagnosis, N (%)	45 (3.7%)	96 (2.9%)	0.2	0.78
				(0.55–1.13)
ED visits with asthma or wheezing diagnoses,	47 (3.9%)	104 (3.2%)	0.3	0.81
N (%)				(0.58–1.16)
SABA purchases, N (%)	499 (41%)	1488 (45%)	0.01	1.19
				(1.04–1.36)
ICS purchases, N (%)	272 (22%)	814 (25%)	0.1	1.14
				(0.98–1.34)
OCS purchases, N (%)	549 (45%)	1564 (48%)	0.15	1.1
				(0.97–1.26)
Asthma-Integrated Diagnosis Index, N (%)	79 (6.5%)	275 (8.4%)	0.04	1.34
				(1.05–1.74)

* Data include diagnoses assigned between 0 and 2 years of age. ** Data provide blood tests conducted between 6 and 18 months of age.

## Data Availability

Restrictions apply to the availability of these data. Data were obtained from the Clalit-Healthcare Service database and are available with permission from the Clalit-Healthcare Service.

## Supplementary Materials

The following supporting information can be downloaded at https://www.mdpi.com/article/10.3390/vaccines12111269/s1. Table S1: ICD codes; Table S2: Incidence rate ratios of clinical outcomes between Prophylaxis (+) and Prophylaxis (–) groups in 1-5 years of age; Table S3: Multivariate logistic regression analysis of healthcare utilization for preschool asthma, comparing Prophylaxis (+) and Prophylaxis (–) groups; Table S4: Multivariate logistic regression analysis of healthcare utilization for preschool asthma, comparing Prophylaxis (+) and Prophylaxis (–) groups—an analysis of children born between July and December.
