# Supplementary material for: The Impact of Palivizumab for Respiratory Syncytial Virus Prophylaxis on Preschool Childhood Asthma
Source: Vaccines (Basel). 2024 Nov 10;12(11):1269. doi: 10.3390/vaccines12111269 (PMC11598595; doi:10.3390/vaccines12111269)
Supplement: Supplementary file 1 [file vaccines-12-01269-s001.zip › vaccines-3245802-supplementary.pdf]

Supplementary Materials

Table S1: ICD codes

Diagnosis	IDC Code
Asthma	493.90 / 493.91 / 493.92 / 493.20 / 492.21 / 493.22 / 493.81 / 493.82 / 493.00 / 493.01 / 493.02 / 493.10 / 493.11 / 493.12
Wheezing	786.07, 744.5

Table S2: Incidence rate ratios of clinical outcomes between Prophylaxis (+) and Prophylaxis (-) groups at 1-5 years of age

Clinical Outcome	Prophylaxis (-) N = 1,216	Prophylaxis (+) N = 3,287	P-value	Incidence Rate Ratio (95% Confidence Intervals)
Admission for RSV bronchiolitis*, N (%)	40 (3.3%)	60 (1.8%)	0.004	0.55 (0.37-0.83)
Allergic rhinitis, N (%)	42 (3.5%)	112 (3.4%)	0.9	0.99 (0.70-1.42)
Atopic dermatitis, N (%)	289 (24%)	843 (26%)	0.2	1.08 (0.95-1.24)
Mean absolute eosinophilic blood ** (±SD)	0.28±0.22	0.3±0.28	0.004	0.55 (0.37-0.83)
ICD-9 codes for asthma diagnosis, N (%)	141 (12%)	435 (13%)	0.14	1.14 (0.95-1.38)
ICD-9 codes for wheezing diagnosis, N (%)	45 (3.7%)	96 (2.9%)	0.2	0.79 (0.56-1.13)
ED visits with asthma or wheezing diagnoses, N (%)	47 (3.9%)	104 (3.2%)	0.3	0.82 (0.58-1.16)
SABA purchases, N (%)	499 (41%)	1,488 (45%)	0.011	1.10 (1.00-1.27)
ICS purchases, N (%)	272 (22%)	814 (25%)	0.10	1.11 (0.97-1.34)
OCS purchases, N (%)	549 (45%)	1,564 (48%)	0.3	1.05 (0.96-1.16)
Asthma-Integrated Diagnosis Index, N (%)	79 (6.5%)	275 (8.4%)	0.038	1.31 (1.04-1.68)

Table S3: Multivariate logistics regression analysis of healthcare utilization for preschool asthma, comparing Prophylaxis (+) and Prophylaxis (-) groups.

Outcome	Variable	Beta	R square[1]	Adjusted Odds Ratio (95% Confidence Interval)	P-value
**ICD-9 codes for asthma diagnosis**	**Prophylaxis (+)**	0.04	0.00	1.04 (0.84-1.30)	0.7
	Atopic dermatitis	0.27		1.32 (1.07-1.61)	0.008
	Absolute eosinophilic count	-0.10		0.91 (0.63-1.28)	0.6
	Birth weight	-0.00		1.00 (1.00-1.00)	0.2
	Cesarean delivery	0.09		1.09 (0.90-1.32)	0.4
**ICD-9 codes for wheezing diagnosis**	**Prophylaxis (+)**	-0.26	0.00	0.77 (0.52-1.16)	0.2
	Atopic dermatitis	0.17		1.19 (0.78-1.75)	0.4
	Absolute eosinophilic count	0.20		1.22 (0.61-2.16)	0.5
	Birth weight	0.00		1.00 (1.00-1.00)	0.4
	Cesarean delivery	0.1		1.11 (0.76-1.62)	0.6
**ED visits for asthma or wheezing**	**Prophylaxis (+)**	-0.24	0.00	0.79 (0.54-1.17)	0.2
	Atopic dermatitis	0.13		1.14 (0.77-1.67)	0.5
	Absolute eosinophilic count	0.08		1.08 (0.54-1.92)	0.8
	Birth weight	-0.00		1.00 (1.00-1.00)	0.8
	Cesarean delivery	-0.14		0.87 (0.60-1.24)	0.4
**SABA purchases**	**Prophylaxis (+)**	0.13	0.03	1.14 (0.98-1.32)	0.084
	Atopic dermatitis	0.51		1.67 (1.45-1.93)	<0.001
	Absolute eosinophilic count	0.26		1.29 (1.02-1.65)	0.034
	Birth weight	-0.00		1.00 (1.00-1.00)	0.7
	Cesarean delivery	0.36		1.43 (1.25-1.63)	<0.001
**ICS purchases**	**Prophylaxis (+)**	0.10	0.02	1.11 (0.93-1.32)	0.3

Outcome	Variable	Beta	R square[1]	Adjusted Odds Ratio (95% Confidence Interval)	P-value
	Atopic dermatitis	0.42		1.52 (1.30-1.79)	<0.001
	Absolute eosinophilic count	0.02		1.02 (0.77-1.33)	0.9
	Birth weight	0.00		1.00 (1.00-1.00)	0.6
	Cesarean delivery	0.39		1.47 (1.26-1.72)	<0.001
**OCS purchases**	**Prophylaxis (+)**	0.09	0.00	1.09 (0.94-1.26)	0.3
	Atopic dermatitis	0.1		1.11 (0.96-1.28)	0.2
	Absolute eosinophilic count	-0.15		0.86 (0.68-1.09)	0.2
	Birth weight	0.00		1.00 (1.00-1.00)	0.7
	Cesarean delivery	0.07		1.08 (0.95-1.23)	0.3
**Asthma-Integrated Diagnostic Index**	**Prophylaxis (+)**	0.16	0.01	1.18 (0.90-1.55)	0.2
	Atopic dermatitis	0.50		1.66 (1.31-2.09)	<0.001
	Absolute eosinophilic count	0.06		1.07 (0.70-1.56)	0.7
	Birth weight	-0.00		1.00 (1.00-1.00)	0.2
	Cesarean delivery	0.13		1.14 (0.91-1.45)	0.3

Table S4: Multivariate logistics regression analysis of healthcare utilization for preschool asthma, comparing Prophylaxis (+) and Prophylaxis (-) groups – an analysis of children born between July and December.

Outcomes	Characteristics	Beta	R square[1]	Adjusted Odds Ratio (95% Confidence Interval)	P-value
**ICD-9 codes for asthma diagnosis**	**Prophylaxis (+)**	0.02	0.01	1.02 (0.74-1.44)	0.9
	Atopic dermatitis	0.16		1.17 (0.89-1.53)	0.2
	Absolute eosinophilic count	-0.11		0.89 (0.56-1.37)	0.6
	Birth weight	-0.00		1.00 (1.00-1.00)	0.043
	Cesarean delivery	0.19		1.21 (0.94-1.57)	0.14
**ICD-9 codes for wheezing diagnosis**	**Prophylaxis (+)**	-0.36	0.01	0.70 (0.40-1.29)	0.2
	Atopic dermatitis	0.43		1.53 (0.91-2.52)	0.10
	Absolute eosinophilic count	0.12		1.13 (0.45-2.31)	0.8
	Birth weight	0.00		1.00 (1.00-1.00)	0.6
	Cesarean delivery	0.02		1.02 (0.62-1.69)	>0.9
**ED visits for asthma or wheezing**	**Prophylaxis (+)**	-0.30	0.00	0.74 (0.43-1.35)	0.3
	Atopic dermatitis	0.00		1.00 (0.58-1.66)	>0.9
	Absolute eosinophilic count	0.15		1.16 (0.49-2.27)	0.7
	Birth weight	-0.00		1.00 (1.00-1.00)	0.5
	Cesarean delivery	-0.26		0.77 (0.48-1.24)	0.3
**SABA purchases**	**Prophylaxis (+)**	0.11	0.03	1.12 (0.89- 1.40)	0.4
	Atopic dermatitis	0.46		1.59 (1.31-1.93)	<0.001
	Absolute eosinophilic count	0.20		1.22 (0.90-1.67)	0.2
	Birth weight	-0.00		1.00 (1.00-1.00)	0.3
	Cesarean delivery	0.43		1.54 (1.29-1.84)	<0.001
**ICS purchases**	**Prophylaxis (+)**	0.17	0.02	1.18 (0.90-1.56)	0.2

	Atopic dermatitis	0.34		1.41 (1.14-1.75)	0.002
	Eosinophils levels abs	0.08		1.08 (0.76-1.51)	0.7
	Birth weight	-0.00		1.00 (1.00-1.00)	0.11
	Cesarean delivery	0.34		1.41 (1.15-1.74)	0.001
**OCS purchases**	**Prophylaxis (+)**	0.04	0.00	1.04 (0.83-1.30)	0.7
	Atopic dermatitis	0.01		1.01 (0.83-1.22)	0.9
	Absolute eosinophilic count	-0.34		0.72 (0.52-0.97)	0.034
	Birth weight	0.00		1.00 (1.00-1.00)	0.9
	Cesarean delivery	0.16		1.17 (0.98-1.39)	0.076
**Asthma-Integrated Diagnostic Index**	**Prophylaxis (+)**	-0.02	0.02	0.98 (0.66-1.50)	>0.9
	Atopic dermatitis	0.43		1.54 (1.13-2.10)	0.009
	Absolute eosinophilic count	0.06		1.06 (0.62-1.68)	0.8
	Birth weight	-0.00		1.00 (1.00-1.00)	0.007
	Cesarean delivery	0.13		1.13 (0.83-1.55)	0.4
